# How do patients with borderline personality disorder experience Distress Tolerance Skills in the context of dialectical behavioral therapy?—A qualitative study

**DOI:** 10.1371/journal.pone.0252403

**Published:** 2021-06-15

**Authors:** Anja Schaich, Diana Braakmann, Mirco Rogg, Clara Meine, Julia Ambrosch, Nele Assmann, Stefan Borgwardt, Ulrich Schweiger, Eva Fassbinder

**Affiliations:** 1 Department of Psychiatry and Psychotherapy, University of Lübeck, Lübeck, Germany; 2 Department of Psychiatry and Psychotherapy, Christian-Albrechts-Universität zu Kiel, Kiel, Germany; University of Bern, SWITZERLAND

## Abstract

Distress Tolerance Skills (DTS) are an important component of Dialectical Behavioral Therapy (DBT), a therapy method frequently used for treating patients with Borderline Personality Disorder (BPD). However, little is known about how DTS-training is experienced by individuals with BPD. The aim of this study was to explore BPD patients’ experiences with receiving DTS-training. Qualitative data were collected through semi-structured interviews with 24 individuals (87.5% females) with a primary diagnosis of BPD who received DTS-training in the context of 18 months of DBT treatment. Interview data were analyzed following the procedures of qualitative content analysis. Participants reported various effects of DTS including an immediate reduction of tension. Patients perceived DTS as a tool to cope with difficult interpersonal situations and emergencies and stated that this helped them to feel stable, safe and self-confident. Patients reported difficulties during the initial engagement, the learning process and the application of DTS as well as various facilitating factors. Implications of the findings for further research and for optimizing DTS-training in clinical practice are discussed.

## Introduction

Dialectical Behavior Therapy (DBT) was developed by Marsha Linehan [1] and is one of the most profoundly applied and studied treatment approaches for patients with Borderline Personality Disorder (BPD) [2]. DBT has proven effective at reducing suicidality, self-injury and impulsive behaviors as well as emergency room visits and inpatient admissions in several randomized controlled trials (RCTs) [2–5].

In DBT, BPD is seen as a disorder of the emotion regulation system in which most of the BPD-symptoms (such as self-injury, suicidality, dissociation, substance abuse) are viewed as dysfunctional attempts to deal with emotional distress. Therefore, the main focus of the DBT-treatment is on teaching the patients more adequate skills to regulate their emotional distress and to integrate those skills in their everyday life [1]. Neacsiu et al. [6] found that DBT treatment indeed leads to an increase in the use of specific behavioral skills and that skill use mediates change for suicidal behavior, depression, and anger control. Zeifman et al. [7] compared 20 weeks of DBT-skills group to an active waitlist control. They found that an improvement in mindfulness and DTS each independently indirectly affected the relationship between DBT skills training and post treatment general psychopathology. Also, in qualitative studies, patients with BPD described DBT skills as helpful for dealing with emotions and reducing problem behavior such as suicidality and self-harm [8]. Quantitative studies, which are important to gain insight into the general effectiveness of DBT and specific DBT techniques, can be complemented by qualitative research when it comes to understanding specific mechanisms that facilitate or hinder the recovery process within DBT treatment. For example, Barnicot et al. [9] investigated factors hindering patients to use skills and ways that helped them overcome these barriers. This kind of research is also especially valuable for practicing therapists as hindering and facilitating factors regarding skill use become apparent.

However, the existing qualitative studies on DBT aimed to examine patients’ general experiences with DBT and skills training and therefore have a rather broad focus on the treatment and the individual skills training components. Little et al. [8] recommend a narrower focus for future qualitative analyses in DBT. Indeed, DBT is a complex treatment with more than 60 skills embedded in four modules (mindfulness, emotion regulation, interpersonal effectiveness and distress tolerance skills) and various therapeutic techniques. Therefore, an investigation of patients’ experiences with the specific DBT modules and technique is necessary to gain more detailed information.

For this reason, we started a qualitative research series on the experiences of BPD patients with specific elements and techniques of DBT (Distress Tolerance Skills Training (DTS), Opposite Action etc.). Such information is crucial in order to optimize delivery of specific treatment techniques, overcome patients’ barriers to the acceptance, understanding, training and implementation of specific skills or techniques in their everyday life and by this enhance clinical outcome and reduce drop-out of treatment. In this article, we will present the results of one part of our qualitative research series in detail, namely the BPD patients’ experiences with one skills training module, Distress Tolerance Skills (DTS) training. Although it is of course important to investigate all components of DBT (e.g. such as the other skill training modules: mindfulness, interpersonal effectiveness or emotion regulation skills), in this article we started our qualitative research series by focusing on analyzing patients’ experiences with DTS.

DTS-training provides skills that are ‘concerned with tolerating and surviving crises […] and with accepting life as it is at the moment’ [1]. They are applied to help endure and accept the tension that is typical for patients with BPD. DST can be divided into crisis survival skills and reality acceptance skills. Crisis survival skills provide short-term solutions that help patients to endure difficult situations in order to prevent dysfunctional behavior such as self-injury. The goal of reality acceptance skills is to ‘reduce suffering and increase freedom when painful facts cannot be changed’ [1]. DTS aim to enable patients to reach an ability to act, to face the perception of their current emotional state and apply emotional regulation skills if necessary.

The present study used thematic analysis of qualitative interviews with BPD patients receiving DTS-training in the scope of a DBT treatment to explore patients’ general experiences with DTS training. In particular, we aimed to gain a better understanding of:

- factors patients perceived as hindering and facilitating when engaging in, learning and applying DTS and
- patients’ perception of the short-term and long-term effects of DTS.

## Materials and methods

### Recruitment

The sample consisted of out-patients recruited from the DBT treatment condition of an RCT comparing DBT with Schema therapy. This study was conducted at the Department of Psychiatry and Psychotherapy at Lübeck University, Germany [10]. Participants aged between 18 and 65 years were considered eligible if they had a primary diagnosis of BPD. In the PRO*BPD minimal exclusion criteria were used and BPD patients with various comorbid disorders and high BPD severity were included. However, a psychotic disorder and current severe substance dependence that necessitates detoxification treatment were considered as exclusion criteria. More information on recruitment, diagnostics and procedures of the PRO*BPD trial can be found in the study protocol [10].

Consecutively recruited participants were included if they have had at least four months of DBT and gave informed consent to participate in the qualitative interview. All patients received a DBT treatment program with one individual therapy session and one group therapy session per week. The group therapy session was given by two therapists. For some of the patients one of the group therapists was also the individual therapist, for most of the patients their group therapists and their individual therapists were not the same persons.

### Procedure

Two of the authors with broad experience in the research and treatment of BPD (EF, US) developed a semi-structured interview (see Table 1 for the parts of the interview relevant to the research questions of this study; see S1 File for the German original). The whole interview addressed participants’ experiences with two different elements of DBT (opposite action and DTS).

**Table 1:** Qualitative interview on components of dialectical behavior therapy–Distress Tolerance Skills.

We are now going to focus on Distress Tolerance Skills 1. Name the most important distress tolerance skills for you (max. 5). 2. What experience have you had so far with the application of distress tolerance skills? 3. How often do you use distress tolerance skills approximately?*(e*.*g*. *several times a day*, *1x/a day*, *1x/a week)* 4. In which situations do you use them? 5. What effects do you notice when you use distress tolerance skills?(*favorable and unfavorable effects*, *short-term and long-term*?*)* 6. How content are you with the effect of distress tolerance skills? 7. How do you explain the effect of distress tolerance skills on yourself? 8. What are hindering factors for you in using distress tolerance skills? 9. What supports you in using stress tolerance skills? 10. Is there anything you would like to say to therapists who teach stress tolerance skills? Was there something you found helpful or unhelpful? Was there something that you would have liked to see more?

After a few questions to get some descriptive information on DTS use, the interview started with open questions about patients’ personal experiences with DTS on which the participants were free to elaborate. If not brought up during free speech, follow-up questions on specific topics were asked (see italics in Table 1). The interviews were conducted between June 2017 and July 2018 and the interviewers were four graduate students in the field of psychology and one psychologist (CM, JA, TL, LS, ALH). The interviewers were not involved in the treatment of the participants, nor had they information about the study outcome at the time of the interviews.

The interviews lasted between 60 and 120 minutes. All interviews were audio-recorded with a digital recorder and were transcribed using MAXQDA 2018 Standard [11]. Transcription was conducted following the protocol of Dresing and Pehl [12].

Written informed consent following verbal and written explanation of the study was provided by all of the participants. The research protocol and amendments were approved by the Ethics Committee of the University of Lübeck (reference number 13–005).

### Data analysis

The data of the interview were analyzed following the procedure of qualitative content analysis [13, 14] using the MAXQDA software [11].

The development of a category system is considered central to the procedure of qualitative content analysis. An inductive approach was taken to the analysis. As a first step in the analysis, five interviews with participants with different characteristics (regarding gender, age and number of treatment sessions) were selected. On the basis of these five interviews, a preliminary category system was generated: Two of the authors (CM and JA) rated these five interviews independently, extracted meaningful passages and attributed codes to relevant text passages.

The definition of the units of the analysis was defined as follows: code unit: every context-related expression of the participant, including nonverbal expressions; context unit: one passage until change of speakers; analysis unit: all interviews available. Subsequently, selection criteria were defined using the research questions. The five interviews were processed for each of the research questions separately by extracting relevant passages that came across as meaningful. A coding frame was developed for transparence and traceability of this process. Subsequently, the extracted passages were paraphrased, and afterwards generalization of the paraphrases took place across all of the five interviews by keeping a low abstraction level.

In a next step of the data reduction, meaningless paraphrases were deleted, paraphrases with the same meaning were summarized and paraphrases with similar meanings were expressed by a new statement [13]. Simultaneously, it was verified whether the actual statement of the patient was captured and whether the text passage was of relevance to the research question. The hereby developed categories were sorted further, summarized or divided if necessary and brought into a hierarchical order. The process of paraphrasing, generalizing and reducing was conducted by two authors (CM and JA) to provide the highest possible extent of objectivity and reliability and was recorded for documentation purposes.

After category development, the two coders met for discussion and a consensual development of a category system. The category system was then presented and discussed in an expert group (including US, MR and DB). Based on the suggestions of the expert group, adaptations of the category system were made, and the remaining 19 interviews were analyzed. After this analysis, in consultation with the expert group, a final adaptation of the category system was made by one of the raters (CM) in which categories were summarized and new categories were integrated if adequate. The results are presented below. In the Results section the phenomenological description of the data is presented, which is closely based on the statements of the interviewed participants. The Discussion section presents the interpretation and critical reflection of patients’ statements as well as conclusions for clinical practice and further research.

## Results

### Sample characteristics

The total number of participants included in the qualitative analysis was 24 (22 female). The mean age was 37.50 (SD = 12. 71). The mean symptom level measured by the Borderline Personality Disorder Symptom Inventory (BPDSI) at baseline was 33.89 (SD = 8.51). The mean number of comorbid axis I and II diagnoses assessed with the Structured Clinical Interview for DSM-IV (SCID I and II) was 4.50 (SD = 1.72) and 1.21 (SD = .93), respectively. The interview participants did not differ from the participants in the DBT condition of the RCT regarding gender (exact *p* = .371), age (U = 553.500, *z* = -1.553, *p* = .120), BPDSI (U = 665.000, *z* = -.432, *p* = .666) as well as number of comorbid SCID I (U = 588.000, *z* = -1.226, *p* = .220) and SCID II (U = 670.500, *z* = -.401, *p* = .688) diagnoses. See Table 2 for demographic and clinical characteristics.

**Table 2:** Demographical and clinical characteristics of the interview patients and patients in the DBT condition of the RCT.

	Interview patients n = 24	DBT condition RCT patients n = 83
**Age**, mean (SD)	37.50 (12.71)	33.17 (15.58)
**BPDSI,** mean (SD)	33.89 (8.51)	33.68 (8.99)
**Gender**, nr (%)		
Male	3 (12.5)	17 (20.5)
Female	21 (87.5)	66 (79.5)
**Highest education level**, nr (%)		
None	1 (4.2)	4 (4.9)
Special needs school	1 (4.2)	1 (1.2)
Secondary school (9 years)	2 (8.3)	15 (18.3)
Secondary school (10 years)	11 (45.8)	30 (36.6)
Secondary school (12 years)	0	3 (3.7)
Secondary school (13 years)	3 (12.5)	10 (12.2)
Professional school	5 (20.8)	13 (15.9)
College degree	0	2 (2.4)
University degree	1 (4.2)	3 (3.7)
**Employment status**, nr (%)		
Homemaker	1 (4.2)	4 (4.9)
Unpaid word (e.g. honorary post)	1 (4.2)	1 (1.2)
Employed	1 (4.2)	7 (8.5)
Unemployed	1 (4.2)	2 (2.4)
Incapacitated for work	20 (83.3)	56 (68.3)
Retired	0	1 (1.2)
**Comorbid disorders (DSM-IV)**, nr (%)		
Axis I		
Affective disorders	20 (83.3)	59 (71.1)
Substance disorders	5 (20.8)	24 (28.9)
Anxiety disorders	23 (95.8)	74 (89.2)
Somatoform disorders	2 (8.3)	13.3 (13.3)
Eating disorders	12 (50.0)	41 (49.4)
Axis II		
Avoidant personality disorder	12 (50.0)	39 (47.0)
Obsessive compulsive personality disorder	8 (33.3)	27 (32.5)
Dependent personality disorder	3 (12.5)	14 (16.9)
Paranoid personality disorder	3 (12.5)	17 (20.5)
Schizotypal personality disorder	1 (4.2)	3 (3.6)
Histrionic personality disorder	3 (12.5)	9 (10.8)
Narcissistic personality disorder	1 (4.2)	4 (4.8)

Abbreviations: BPDSI = Borderline Personality Disorder Symptom Inventory

In the year before their participation in the RCT, the interview participants had a mean number of inpatient treatment days of 55 (SD = 49.26) and mean admission to an emergency room due to self-harm or suicide attempt of 1.13 (SD = 3.23). At the time of the interview, one patient had already completed 18 months of treatment and 23 participants had already received between four and seventeen months of therapy (M = 8.78, SD = 3.55). One participant dropped out of therapy after the qualitative interview at a later stage of treatment. As DTS training is one of the first skills training components taught in DBT, all of the participants had DTS-training and therefore at least some experience with DTS.

Table 3 gives descriptive information about the use of DTS mentioned by the participants in this study.

**Table 3:** Background information on the use of Distress Tolerance Skills (N = 24).

	n	%
**Frequency of DTS use**		
Every day	6	25
Several times a week	11	46
1–3 x per month	3	13
< 1 x per month	3	13
Kind of DTS used*		
Distracting with wise mind ACCEPTS	23	96
Self-soothing	15	63
TIP Skills	13	54
Improving the moment	4	17
Pros and Cons	2	8
Reality Acceptance Skills	1	4
**Situations in which DTS are used**		
High level of tension	2	21
Interpersonal interactions	14	58
Intense emotions	8	33
Public transportation	4	17
Public situations	3	13
Nervousness before an appointment	3	13
Driving a car	4	17
Everyday problems	1	4
Being alone	1	4
Difficulty falling asleep	1	4

Abbreviations: DTS = Distress Tolerance Skills; ACCEPT = There are seven sets of distraction skills. The word ACCEPTS is a mnemonic for these strategies: Activities (discordant to the negative emotion), Contributing, Comparisons, Emotions (opposite to the current negative emotion), Pushing away from the situation, Thoughts, and Sensations; TIP Skills = The TIP skills can be used to change body chemistry quickly, so as to counteract disabling emotional arousal. TIP is a mnemonic for Temperature, Intense exercise, Paced breathing, and Paired muscle relaxation.

*Patients statements about the DTS they used were sorted into the DTS subgroups by one of the authors (CM)

### Qualitative content analysis

The thematic analysis of patients’ reports revealed that participants clearly emphasized the process they had to go through in order to master DTS and resulted in four key domains reflecting that process: Initial engagement in DTS (Domain A), learning DTS (Domain B), applying DTS (Domain B) and perceiving the effects of DTS (Domain D). Table 4 gives an overview on the derived category system with main categories and themes.

**Table 4:** Category system (N = 24).

Domains / Themes			n
**A. Initial engagement in Distress Tolerance Skills**			
A1.		Accepting Distress Tolerance Skills is the first step	24 (100%)
**B. Learning Distress Tolerance Skills**			
B1.		Developing individually suitable Distress Tolerance Skills is crucial	21 (88%)
B2.		You have to practice Distress Tolerance Skills	22 (96%)
B3.		Specific aspects of the therapeutic relationship and therapist behaviors can facilitate the successful implementation of Distress Tolerance Skills	18 (75%)
**C. Applying Distress Tolerance Skills**			
C1.	Distress Tolerance Skills are difficult to perform in front of other people		20 (83%)
C2.	To use Distress Tolerance Skills, they must be mentally and practically available		19 (79%)
C3.	The effectiveness of Distress Tolerance Skills is dependent on the level of tension		24 (100%)
C4.	It is important to apply Distress Tolerance Skills mindfully		15 (63%)
**D. Perceiving the effects of Distress Tolerance Skills**			
D1.	Incorrectly applied Distress Tolerance Skills can be harmful		10 (42%)
D2.	Distress Tolerance Skills immediately reduce tension		24 (100%)
D3.	Distress Tolerance Skills stabilize in the long run		23 (96%)
D4.	The effect of Distress Tolerance Skills changes over time		5 (21%)
D5.	Distress Tolerance Skills are the first step in therapy		14 (58%)

#### Domain A: Initial engagement in Distress Tolerance Skills

This domain reflects patients’ first involvement with DTS. In order to apply DTS, patients first need to accept the technique and the underlying theoretical model.

*Theme A1*: *Accepting Distress Tolerance Skills is the first step*. All 24 patients mentioned the acceptance of DTS to be an integral part of the learning process. This domain was linked to three subthemes with two sub-subthemes each.

Subtheme A1.1. The initial engagement in DTS is difficult. Twenty-two (92%) patients reported having difficulties to engage in DTS: *“In the beginning*, *it is difficult to get engaged in it*. *Well*, *what*, *for me it was difficult*, *to get engaged”* (P21). Most of the patients reported having encountered these difficulties at the beginning and to have overcome them at least partially during the course of the treatment.

a) Lack of willingness: Standing in my own way: Sixteen patients (67%) reported not wanting to use DTS. Nine of them (38%) experienced an inner resistance to use skills. They felt it was unfair and frustrating that they have to use skills: *“[…] Why me*, *why do I have to do that*? *And this railing against one’s fate*.*”* (P15). Another problem was bringing up the motivation to use DTS: *“But sometimes*, *the motivation is not*, *ehm*, *there*. *That you get up now and get this spikey ball now*.*”* (P20). Also some participants explained that sometimes they just wanted to be impulsive or have angry feelings and experienced DTS as restricting their freedom: *“[…] sometimes I don’t even WANT to use it*, *that I just want to be stubborn or angry for once […]*.*”* (P16).

Eleven patients (46%) had difficulties to see that they needed DTS at all. Some did not recognize their own distress: *“[…] if the body terrorizes you*, *at some point you get used to it and […] I never thought I have a problem*.*”* (P9). Others found DTS ridiculous: *“[…] so my mentality was*: *‘What does he want with these stupid skills*?*’ And that was all kid’s stuff […]”* (P15). Those patients reported that they did not believe DTS could work: *“It doesn’t matter*, *such bullshit*, *what’s with that*? *Eh*? *So*, *it’s no use anyway*.*”* (P2).

b) Tension is regulated by problem behavior better and faster: Nine patients (38%) reported regulating unpleasant tension by self-harming or impulsive behavior: *“Something like that is still there*, *like that I still use emotional eating and shopping to regulate tension” (P9)*. The patients explained this by the fact that these behaviors were already familiar and did not need to be developed first: *“And to simply get rid of it in another way is just like the familiar thing and therefore somehow easier”* (P10). In addition, patients stated that these behaviors often worked better than DTS: *“These negative behaviors*, *to continue those*. *Because they do work so wonderfully*.*”* (P19). One patient reported being afraid to give up her problem behavior: *“[…] simply the fear behind it*: *What comes next*? *So*, *and I would rather have the ‘smoking’-a ‘problem behavior’- as a skill than to know what comes next”* (P4).

Subtheme A1.2. Desire to change helps with acceptance of DTS. Sixteen patients (67%) reported engaging in DTS with the aim of changing their situation or behavior: *“And I do want a change*. *So*, *if you don’t have that*, *then you don’t need to join any DBT group*. *So*, *I want the change*.*”* (P7).

a) I want to change my problem behavior: Thirteen patients (54%) mentioned the reduction of problem behavior as a goal. These patients reported wanting to reduce their harmful behavior and *“seek alternative ways to regulate my tension”* (P6). Six patients (25%) described problem behavior as a short-term solution for high levels of tension but regarded the behavior as harmful in the long run. For example, the long-term burden was reflected in the high costs: *“In the past I used to freak out and hit people or something and I paid a lot of money for it”* (P23) and difficulties with social integration: *“Aggression is something […]where I have already done a lot of damage by ending relationships or employment relationships and stuff*.*”* (P9). For three patients (13%), the concern that others might notice their problem behavior formed the motivation to change: *“[…] the fear of having*, *um*, *an injury*, *which could have made others aware again of what I have done […]*.*”* (P18).

b) I want to live with a lower level of tension: Seven patients (29%) expressed the desire to feel less tension in their everyday life. This was related to the hope of an improvement in their cognitive performance: *“Well*, *it is*, *I have an amazing memory*, *I can really remember a lot*. *But because my borderline-douche eats up so much energy*, *my brain only remembers half of it*.*”* (P7) as well as to a more stable social environment: *“[…] such a normal social life*, *which I do wish for*, *is simply hardly possible with that*.*”* (P10)

Subtheme A1.3. The group facilitates the acceptance of DTS. Twelve patients (50%) mentioned the group to have helped them accept DTS. This subtheme was linked to two sub-subthemes.

a) I am not alone with my problems: Nine patients (38%) experienced it as helpful to not feel alone: *“Well*, *group therapy is especially important for me to realize that I am not alone”* (P11). Since other patients were struggling with similar problems, the patients felt less like outsiders: *“That you don’t always think you are an alien*. *[…] totally crazy guy*, *but that you just see that others have similar problems*. *That’s also comforting sometimes*.*”* (P15) and they felt understood: *“[…] that I realize that there are people here who understand what I am talking about*. *They feel the same way*.*”* (P9)

b) New perspectives are presented: The acquisition of new perspectives on DTS was described by five patients (21%) as helpful in the learning process: *“So*, *it just helps at times*, *the perspective then also changes sometimes*.*”* (P19).

#### Domain B: Learning Distress Tolerance Skills

After accepting DTS, patients need to choose their individually suitable DTS and practice them intensively in order to be able to successfully apply them. This learning process is facilitated by specific factors.

*Theme B1*: *Developing individually suitable Distress Tolerance Skills is crucial*. Twenty-one patients (88%) commented on the search for DTS that worked for them: *“Just finding out for yourself […] what works best right now”* (P16). This domain was linked to three subthemes.

Subtheme B1.1. DTS work differently for everybody. Twelve patients (50%) emphasized that “*every person needs different skills*.*”* (P9) and one has to adapt DTS *“to themselves*. *Because it doesn’t work the same for everyone*.*”* (P21). While for some patients, sensory stimuli such as *“tasting something hot”* (P21) worked best, these did not reach other patients at all: *“Smelling ammonia and all that*, *that does not help me at all*. *Or sucking on a spicy piece of candy or something like that”* (P16) but they responded better to e.g. cognitive skills.

Subtheme B1.2. Overload: How do I find my DTS?. The search for the right DTS was found to be extremely difficult by 15 patients (63%): *“How do I actually find out what is right for me*? *That’s difficult*.*”* (P17). This subtheme was linked to three sub-subthemes.

a) Which DTS do I use when and how exactly: Ten patients (42%) emphasized the importance of learning the theory behind DTS and how to apply them correctly: “*Because I need to know in which situations I do that*. *Why I do it*. *That I have to do it with mindfulness*.*”* (P21). These patients felt insecure: *“Which skill do I use now and how and for what*?*”*(P16). They expressed the need to learn *“which skill could possibly work in which situation*.*”* (P18) and wished for precise instructions on how to use them: *“How do I actually apply it correctly*? *What exactly am I doing*? *Step by Step*. *This is something I need*.*”* (P17).

b) There is a wide range of DTS: Six patients (25%) stated that the wide range of DTS was overwhelming: *“There are an insane number of skills”* (P9). Ten patients (42%) spoke of the need to try out many DTS to find suitable ones: *“[…] actually*, *just trying the whole range just like that*, *I think*, *something I like*.*”* (P3). This meant that a lot of time and resources had to be invested in the search. However, patients also stated that these many DTS formed *“a huge treasure trove in the end*, *there is always something that is yet to discover*, *something that is good for you*.*”* (P15).

c) At high levels of tension, the receptiveness is reduced: Three patients (13%) reported that they had trouble internalizing DTS due to their tension: “*I often retain only half of it somehow and yes*, *that is kind of a dangerous half-knowledge then*.*”* (P7).

Subtheme B1.3. Joint learning of the DTS helps to find my own DTS. Fifteen patients (63%) commented on the joint learning process of DTS with the group members and the therapists. This subtheme was linked to three sub-subthemes.

a) The possibility of exchanging experiences with the group: Twelve patients (50%) said that they found the exchange of experiences with other group members very helpful: *“Well*, *I found it helpful that we*, *like*, *exchanged experiences*.*”* (P10). The patients received a wide variety of information during group sessions, because *“there are many different people in the group*, *with already many different experiences with DBT”* (P5). Therefore, patients reported that they were able to learn a lot: *“So yes*, *you learn something from each individual group member*, *I find that so incredibly important”* (P15).

b) Joint development of DTS in a practical way: Eight patients (33%) described practical exercises during group sessions as helpful: *“[…] and we just tried out something together*, *which could be the right thing*. *[…] that was very helpful*.*”* (P15). Through such practical experience the DTS were consolidated: *“[…] then that would […] stick in your head more*.*”* (P17). Four patients wished for a *“practical skills session”* (P13) which should take place regularly in group therapy.

c) The possibility of applying the other patients’ DTS myself: Six patients (25%) saw the exchange between group members as an opportunity to *“learn new things*, *skills”* (P11): *“‘Oh*, *I tried this and that or I use this and that and so on*.*’ And you hear that*, *or get to see that*, *can test it*, *can check*: *yes*, *that could be something for me*, *or not*. *I can decide that for myself*.*”* (P17).

*Theme B2*: *You have to practice Distress Tolerance Skills*. Twenty-two patients (96%) made statements on practicing DTS. Almost all of the patients were of the opinion that DTS *“really needs to be practiced*, *because it’s not easy at all*.*”* (P2). Patients’ statements could be linked to three subthemes.

Subtheme B2.1. Personal responsibility: I have to put in the work. Seven patients (30%) saw it as their own responsibility to learn DTS: *“It’s up to me now to change*, *[…]and no one else*.*”* (P23). Two patients explained that in order to accomplish change, they must choose the *“hard way”* (P5): *“[…] these exercises eventually lead to reaching one’s goals*.*”* (P17).

Subtheme B2.2. Practicing DTS is a long, tedious process. Twenty-two patients (96%) stated that the learning process is *“not the most comfortable way”* (P10). They described it as a *“slow process”* (P5) and found it *“strenuous”* (P11) or *“extremely annoying”* (P24). Patients reported that success did become apparent, but only after some time: *“Well*, *that was a long learning process*, *but it has improved*.*”* (P15). This subtheme was linked to three sub-subthemes.

a) DTS do not always work: Seventeen patients (74%) mentioned frustration because DTS didn’t work at first: *“Well*, *that’s just frustrating*. *And I’m annoyed about the skills*, *about the skills and about me […]*.” (P17). This experience could lead to uncertainty and the feeling of being a failure in some patients: “*[…] you*, *like*, *think you are a particularly stupid Borderliner*.*”* (P9). In some patients the tension would even increase. In patients with the *“expectation that it works right away”* (P8) this would be particularly difficult because they tend to give up DTS altogether *“[…] that I say*: *doesn’t work*, *didn’t help me”* (P8).

b) Automate DTS so that they can be used in everyday life: Thirteen patients (57%) stated that the training process ideally ends in the automated application of DTS: *“[…*.*] that it gets automated*. *That would be*, *say*, *reaching the goal 100%”* (P23). Patients stated they needed to internalize DTS to such an extent *“that the body automatically demands it*, *even if you are no longer able to think about it with your head*.*”* (P1). To achieve this goal according to the patients, a lot of training is necessary: *“Well*, *it is a skill*, *[…] that you really have to try again and again […] until it becomes second nature”* (P16). Many patients reported that they hadn’t reached this point yet: *“Well*, *I still think I am at the very beginning*. *I do have already learned a lot*, *but I have to say that it is very difficult to implement this in everyday life*.*”* (P2).

c) Practice for emergencies in a state of low tension is necessary: Six patients (26%) felt that it was important to practice DTS in a state of low tension to become familiar with it: *“What I found helpful was that we tried out DTS in states of low tension*. *[…] That we were already trying out DTS then to see what kind of effects they have*, *how I have to use them*.*”* (P21).

Subtheme B2.3. DTS can only be really understood through personal experience in an emergency situation. Six patients (26%) emphasized that practical experience with DTS is necessary to really understand it: *“[…] so*, *you really have to experience that it does something to you*. *That it takes off the tension and you get your head clear again and*, *um*, *if you have had this experience once or twice*, *then you kind of understand the purpose of the skill*. *Only then*.*”* (P15).

*Theme B3*: *Specific aspects of the therapeutic relationship and therapist behaviors can facilitate the successful implementation of Distress Tolerance Skills*. Eighteen patients (75%) described various factors that facilitated the understanding of DTS and motivated them to engage in it. These factors could be linked to two subthemes.

Subtheme B3.1. Ensure the understanding of the content of DTS. Fourteen patients (58%) pointed out how important it is to enable a good understanding of DTS.

a) Adapting the pace of teaching to the patients: Eight patients (33%) emphasized that the implementation should be neither too fast nor too slow. The patients felt that an important task of the therapists was to ensure that all patients had a good understanding of the DTS: *“[…] did really everybody understand it*. *That the therapist himself has this intuition*: *[…] Can he really use this as a tool*?*”* (P22). Patients reported that excessive demands can lead to frustration but that there can also be a feeling of being underchallenged when the introduction to the technique is perceived as *“sluggish”* (P7): *“[…] is presented as if you are in kindergarten*. *Like that*, *that sometimes makes me go nuts”* (P3).

b) Providing information on the benefits and limitations of DTS in advance: Seven participants (29%) demanded a comprehensive explanation of DTS and its strengths and limitations in order to prevent misunderstandings. They found it helpful to know that DTS are only tools for the moment and not the solution to the underlying problems: *“[…] that the DTS would not eliminate the problem*. *That is often*, *like*, *the assumption*. *This is like a pill then*. *Then I take this and then it works again*. *It’s just not like that*.*”* (P10). In addition, some patients mentioned that they experienced less frustration during the learning process because they were prepared for upcoming difficulties: *“[…] it was also pointed out […] that it just doesn’t work the first time*. *And that’s how we know that and we can deal with it accordingly*.*”* (P23).

c) Providing illustrative examples: Four patients (17%) stated that illustrative examples helped them with the learning process of DTS: They could remember DTS better and were more likely to implement them: *“[…]the best way to really remember it is*, *if you have examples and then you are also*, *I noticed*, *more motivated*, *then you come up with other things yourself as well*.*”* (P17).

Subtheme B3.2. Trust and respect within the therapist-patient relationship. Thirteen patients (54%) highly appreciated the value of a good relationship with their therapists based on mutual understanding and respect on the one hand and a clear leadership by their therapist in difficult situations on the other hand: *“Yes*, *well*, *I can only say that I am so lucky with my therapist because we are on the same wavelength […] And who can also give you good advice on how to deal with certain crises*.*” (P7)*.

a) Clear guidance by therapists helped patients to feel safe: Seven participants (29%) wished for *“a real captain”* (P7) who could *“carry you along”* (P11) during the implementation of DTS. They stated that the therapists should be able to assert themselves in the group: *“No*, *there must be someone who says*: *‘I am the therapist and this is how it’s done and this is it*.*’”* (P7). Patients reported to feel *“safe and secure”* (P16) with such frame conditions.

b) Therapists should attend to the patients: Seven patients (29%) reported that their therapists tried hard to understand them: *“I find it totally helpful that they just keep asking questions*, *but of interest*. *Like*, *not with a raised forefinger*. *But really trying to inquire in order to understand*.*”* (P1). They felt that their needs were addressed in therapy.

c) It is helpful if the patients open up: For five patients (21%), their own attitude was also an important prerequisite: *“Yes*, *well of course the [patient] must also be ready to listen to them*.*”* (P9). Patients explained that they have to open up in order to benefit from the therapy: *“[…] in the meantime*, *I can really talk to the therapist openly about my problems”* (P16), which can sometimes be difficult: *“Yes*, *at the beginning*. *Well*, *it is quite*, *it is quite new in some way*. *You’d like to say something*, *but you still closed up anyway*.*”* (P20).

d) Patients may address uncertainties: Five patients (21%) stated that, during the therapy, they had the possibility to ask questions:”*Well*, *if there are any uncertainties or something like that*, *there is always enough room to ask […]*.*”* (P24). They reported this to be helpful and stated that they got more confident to interrupt the sessions with their questions:”*[…] that we can also ask questions at any time […] no fear or even anxiety anymore*.*”* (P13).

#### Domain C: Applying Distress Tolerance Skills

The application of DTS was associated with various difficulties and constraints that affect the successful use of DTS.

*Theme C1*: *Distress Tolerance Skills are difficult to perform in front of other people*. Twenty patients (83%) reported having difficulties using DTS with other people present. Patients’ statements could be linked to five subthemes.

Subtheme C1.1. Nobody should notice the DTS. For 18 patients (75%) it was important to use their DTS “*without anyone noticing”* (P21). To avoid drawing attention, nine patients (38%) reported to use inconspicuous DTS. Small DTS such as *“this little ring […] in the pocket or hidden” (P9)* or *“cognitive things like […] counting red cars or something*.*”* (P10). Twelve patients (50%) reported a fear of being judged and that they pay a lot of attention to the reactions in their environment when using DTS. They described how other people reacted with incomprehension and that they are worried about being labelled a problem case: *“For example*, *with ammonia*, *you just have to*, *like*, *open the bottle and yes*, *sniff it […] Well*, *I wouldn’t do that in public*, *because I think what it looks like*, *it looks like drug-dependent somehow*.*”* (P13). Patients emphasized the importance to belong: *“You want to be part of society*. *I think for us it’s especially important*, *not to be excluded*.*”* (P11).

Subtheme C1.2. Worrying about burdening someone. Three patients (13%) declared that they did not want to burden others (e.g. friends or own children) by using DTS: “*I just want them to have as little contact with these things as possible and um*, *somehow notice it and say*: *‘Mom*, *what kind of candy are you taking*?*’ Or*, *um*, *‘Why do you have a rubber band around your wrist*?*’”* (P8).

Subtheme C1.3. In some situations, DTS are inappropriate. Five patients (21%) named various situations in which the use of DTS could have negative effects. Work situations were mentioned particularly frequently: *“[…] if you are sitting with the boss or something like that*, *and if then suddenly walking out would be appropriate*.*”* (P18), but also general social situations: *“Yes*, *I don’t feel like smelling a urine cup [with ammonia] on the bus right now*, *for example*. *That is just not very (…) appropriate*.*”* (P11).

Subtheme C1.4. To use DTS, I need quietness. For six patients (25%) the DTS could only work if there was space and peace and quietness: *“Not*, *uh*, *having no room at home where I can do it in peace*, *that’s obstructive for me*.*”* (P15).

Subtheme C1.5. If necessary, I use DTS in public. Only one patient (4%) reported to use DTS in public, if necessary: *“[…] I have no problems with that*. *It’s just*, *at that moment*, *if it is necessary*, *it is necessary*.*”* (P19).

*Theme C2*: *To use Distress Tolerance Skills*, *they must be mentally and practically available*. Nineteen patients (79%) stated that DTS can only be used if they have access to them: *“You just have to remember that then*. *Or have that on you*.*”* (P1). Patients’ statements could be linked to two subthemes.

Subtheme C2.1. I don’t think of DTS when I am in a state of high tension. Sixteen patients (67%) described difficulties with using DTS in states of high tension: *“because under pressure and stress it is simply buried in oblivion*.*”* (P2). Patients reported not to have access to the DTS mentally even if they have them on hand: *“Well*, *personally*, *it didn’t help me practically when I already was at 160*, *uh*, *that I had ampoules with ammonia in my backpack*. *I just didn’t think of them at all*.*”* (P9). Six patients (25%) stated that it was helpful when others reminded them of their DTS: *“Then*, *when my husband says*: *‘Do your skill’*, *he brings me*, *like*, *back to the here and now […]*.*”* (P15).

Subtheme C2.2. I don’t always have my DTS at hand. For 14 patients (59%) it was *“hindering that these are just things that have to be there*.*”* (P17). To be able to use DTS in an emergency, patients would have to always carry them around. Four patients (17%) reported that they sometimes forgot to take their DTS with them: *“Yes*, *well*, *now and then*, *you leave the spikey ball at home or something*.*”* (P16). Some DTS could not be accessed easily: *“Yes*, *and you don’t always have the option of taking a cold shower*.*”* (P20). Four patients (17%) reported using DTS that are *“easy to integrate”* (P19) and *“easy to take with you”* (P21) and therefore minimize problems.

*Theme C3*: *The effectiveness of Distress Tolerance Skills is dependent on the level of tension*. All 24 patients reported having learned that the level of their tension influenced how the DTS worked: *“I only understood much later*, *in group therapy or therapy*, *that the skills are very dependent on what*, *let’s say*, *temperature condition one is in*.*”* (P9). Patients’ statements on this topic could be linked to four subthemes.

Subtheme C3.1. I need to be able to recognize my level of tension. Twenty patients (83%) emphasized that perceiving their level of tension was an important prerequisite: *“So*, *it really has a lot to do with recognizing*, *perceiving yourself*, *at what point are you approximately*?*”* (P9). Having this ability was accomplished only slowly during the treatment: *“[…] it takes a long time*, *right*? *[…] To feel*, *to sense where the tension is at*.*”* (P12). Some patients reported that they still had severe difficulties estimating their own level of tension, while others reported to already have mastered it: *“Yes*, *this tension*, *you have to check it*, *right*? *Um*, *and now I’m actually notice it myself already*.*”* (P15).

Noticing their increase and decrease of tension was perceived as important by nine patients (38%) in order to use the DTS. An early reaction to an increase of tension would ensure *“that I don’t get SO high into the stress”* (P16): *“that I notice more quickly when tension increases and that I have more of*, *like*, *the opportunity to intervene*.*”* (P10). Twelve patients (50%) reported using different DTS depending on their level of tension: “*That MUST be the right tension then*. *So […] there is a skill for every tension […] they have to fit*. *It’s like a puzzle*.*”* (P7).

Subtheme C3.2. Some DTS don’t work in states of extreme tension. Twenty-two patients (92%) described that some DTS would not work in states of high tension: *“[…] and then I put my lavender bottle under my nose and then I calm down a little*. *[…] but that doesn’t work when I am at the top […]*.*”* (P2). Eleven patients (46%) found themselves unable to apply DTS requiring cognitive performance or fine motor skills at high levels of tension.

Stimuli were perceived completely differently by five patients (21%) in high compared to low states of tension: *“In a relaxed state […] ammonia also smells bad to me*. *And pepper plaster is also very bad*. *It’s only*, *[…] when I am really in like such a high-boiling situation […] it doesn’t smell and the pepper plaster doesn’t work*.*”* (P9). Twelve patients (50%) agreed that only a *“strong impulse”* (P10) could reach them in states of high tension: *“That’s*, *um*, *if the tension is so high that you become incapacitated*, *then you need something like*, *like a hammer blow that brings you back and those are of course very intense skills*.*”* (P7). Ammonia and spiciness were named particularly frequently in this regard.

Subtheme C3.3. I need DTS in states of high tension. For 21 patients (88%), DTS were necessary *“when you are out of control”* (P24): *“If my tension is over*, *um*, *60%*, *like*, *I really can’t think any more […]*. *Then*, *um*, *there are several ways to reduce tension*, *these are the DTS*.*”* (P11).

Subtheme C3.4. In lower states of tension, I use mindfulness exercises and emotion regulation. Fifteen patients (65%) described which skills they used in lower states of tension. They mentioned mindfulness skills: *“The lower the area of tension*, *then exercises*, *mindfulness exercises*.*”* (P21) and emotion regulation skills: *“[…] if I find something positive in my brain*, *in my feelings*, *then […] emotion regulation is in the middle*, *well exactly*.*”* (P13).

*Theme C4*: *It is important to apply Distress Tolerance Skills mindfully*. Fifteen patients (63%) explained that they always use DTS in conjunction with mindfulness: *“I think*, *uh*, *mindfulness is certainly the key for me*.*”* (P15). Patients reported that the mindful application of DTS is effective *“because all the senses are concentrated on it at first*.*”* (P3).

#### Domain D: Perceiving the effects of Distress Tolerance Skills

*Theme D1*: *Incorrectly applied Distress Tolerance Skills can be harmful*. Ten patients (42%) talked about the risk of using DTS incorrectly and thereby causing negative consequences. Patients’ statements could be linked to three subthemes.

Subtheme D1.1. Some DTS may generate pressure to perform. Three patients (13%) shared their unpleasant experiences with certain DTS that required some kind of performance. As examples they mentioned *“playing the piano”* and *“learning French vocabulary”* (P15), *“baking a cake”* (P16) and *“doing an animal alphabet”* (P9). They reported that if these DTS fail, they can put even more pressure on the patient instead of reducing the tension: *“It actually is relatively good for me*, *playing the piano then*. *Whereas I have to be careful that I get into this spiral of being pressured to perform*.*”*(P15).

Subtheme D1.2. Applied in an extreme way, a DTS can become a problem behavior. Five patients (21%) warned against using DTS in an excessive way. Very cold stimuli can lead to injuries: *“Of course*, *you have to be careful not to press the cool pack*, *like*, *too hard somehow*, *somewhere*. *You don’t want to get*, *say*, *freezer burn*.*”* (P12) They also warned against the excessive use of spicy stimuli: *“[…] I can tolerate a lot of chili*, *so what is spicy for me*, *other people can’t eat*. *[*…*] so that’s where my gastrointestinal tract is at*. *I don’t think I can do this forever*.*”* (P7). For this reason, patients stated that it must be ensured that the DTS does not become a problem behavior itself: *“Well*, *of course I have to be careful*, *[…] that I don’t go back to extremes/ So 20 kilometers are enough at the moment*, *I’ve been told*. *Not 40 again right away*. *And when I have cycled 30 kilometers*, *[…] And um jogging there and back again*, *then that is problem behavior again*.*”* (P19).

Subtheme D1.3. I should not start avoiding all emotions by using DTS. Seven patients (29%) saw a risk in abusing DTS to avoid emotions: *“[…] I have to be careful that I don’t try to make emotions or unpleasant situations go away by using skills*. *That works*, *too*. *Like*, *when I notice*: *Oh*, *something is unpleasant*. *Well*, *I’m going to go throw some chili and other things*. *That’s just another avoidance strategy*. *It’s a bit of a fine line*, *like*, *yeah*.*”* (P10). According to the patients, this avoidance strategy would prevent further progress *“because some emotions should be allowed*, *that’s just the way it is*.*”* (P19).

*Theme D2*: *Distress Tolerance Skills immediately reduce tension*. All 24 patients described DTS as *“aids and skills that reduce stress*.*”* (P1). The DTS were reported to have a general calming effect: *“Yes*, *simply that I’m getting calmer […]*.*”* (P16). Patients’ statements could be linked to eight subthemes.

Subtheme D2.1. Becoming capable to function again. Eight patients (33%) mentioned to use DTS *“to restore my ability to function*.*”*.

Subtheme D2.2. The tension in the body is reduced. Five patients (21%) described a physical relief after the application of DTS: *“Then you also notice how*, *how it*, *the*, *uh*, *body tension also becomes so relaxed again”*. (P12). The high tension was described by them as very unpleasant: *“[…] I’m really boiling in my head and in my whole body and I think somehow I’m in the middle of hell”* (P13), and that the DTS create *“a moment of well-being”* (P15).

Subtheme D2.3. DTS help to come back to the here and now. Seven patients (29%) reported anti-dissociative effects of DTS: *“[…] brings you out of the highest level of tension to be back in the here and now*.*”* (P21). They stated that they were being brought back *“to earth”* (P3). Some patients stated that DTS helped them resume the perception of themselves: *“that I then also feel myself again*, *also physically*.*”* (P19) and their environment *“that I then had a feeling in what kind of situation I am realistically in*.*”* (P22).

Subtheme D2.4. DST help to think clearly again. Twelve patients (50%) reported that DTS helped them to stop ruminating and *“to think normally again*.*”* (P6). The DTS allowed them to break out of “*this spiral of thoughts”* (P8) and only in a *“reasonably thinking range”* (P16) it was possible for them to reflect on their reactions and to choose better strategies: *“But that’s when I calm down a bit […] and can then with my head*, *think about a strategy for […] for the remaining hours or the next day*.*”* (P19).

Subtheme D2.5. DTS work quickly. Eight patients (33%) considered it important to *“get out of the state of high tension as quickly as possible”* (P21) and described that they succeeded in doing that by using DTS: “*Then you’re completely off the track at first*, *but you get caught up really quickly*. *And then you’re back again*.*”* (P7).

Subtheme D2.6. DTS help to prevent problem behaviors. The avoidance of problem behavior was presented by 17 patients (71%) as an important effect of DTS. The patients saw DTS as a way to regulate their tension *“without harming yourself”* (P6) and were glad to be able to prevent their problem behavior: *“Well*, *for me it was really important now*, *that to get a way of regulation and for example just not get angry so quickly*, *not to freak out so quickly […]*.*”* (P3).

Subtheme D2.7. DTS distract from internal and external distress. Eighteen patients (75%) noticed that they were *“somehow distracted”* (P1) while using DTS. DTS moved their focus off their problems: *“Simply that*, *that the attention is directed elsewhere*, *yes*.*”* (P18). This distraction helped them to endure unpleasant sensations better: *“Then I start like counting things that are in the room and then I try to distract myself from it with these cognitive things*, *so to speak*. *Without wanting to make the feeling go away*, *now*, *just to tone it down*, *so that I can continue to endure the situation*.*”* (P11).

Subtheme D2.8. DTS bring up unpleasant emotions again. Six patients (25%) reported that after the use of DTS they initially experienced unpleasant emotions: *“And most of the time*, *initially*, *it’s more unpleasant after the application of DTS than before*.*”* (P10). As these sensations were blocked previous to the application of the DTS, patients reported feeling overwhelmed by them: *“If*, *when I do them*, *[…] then it also happens that my feelings really explode*.*”* (P2).

*Theme D3*: *Distress Tolerance Skills stabilize in the long run*. The long-term effect of DTS was assessed by 23 patients (96%) as being stabilizing: *“Yes*, *it definitely helps to stay more stable this way*, *like*, *yeah*.*”* (P24). Patients’ statements could be linked to six subthemes.

Subtheme D3.1. Better insight into and acceptance for problems. Sixteen patients (67%) reported that they developed a better understanding of themselves through the use of DTS. They stated that they knew their problem areas: *“[…] this whole thing that I usually perceive as a huge emotional tangle is then easier to distinguish and differentiate*, *[…] only then can I notice which situations trigger what*, *anyway*.*”* (P10). They reported to try to be forgiving of their setbacks and mistakes with the goal of being able to accept themselves better: *“I am working on being able to accept myself*. *I’m on a good path*.*”* (P1).

Subtheme D3.2. States of high tension are less frequent. Eighteen patients (75%) reported that they are now *“quite balanced”* (P5) and would get into states of high tension less frequently: “*That I don’t get SO high in stress anymore […]*.*”* (P16) and that they wouldn’t get into a state of high tension that easily any more: *“In the past*, *I went straight up*, *um*, *today it takes a bit longer*.*”* (P2). This subtheme was linked to three sub-subthemes.

a) DTS are needed less often: Because states of high tension would occur less frequently, eight patients (33%) stated they also needed to use DTS less frequently. They saw this as a sign of their development: *“In the beginning I thought like*, *okay*, *then I have to carry around a stress skill all my life*. *It is also good to notice that it is simply less*. *That when you learn the other things*, *it’s not that necessary anymore*.*”* (P1).

b) DTS are integrated into everyday life: Eight patients (33%) described how they try to *“make regular use of the skills*, *thus to integrate them into the daily routine*.*”* (P8). Two participants described rituals that helped them to a relaxed start of the day: “*I do one thing before work […] and then I am calmed down really*, *and then I go to work*.*”* (P4) while one patient reported to end the day calmly with an DTS: *“Yes*, *but I do meditate like this every evening*. *Well*, *this is a skill that I actually use every day*.*”* (P20).

c) DTS are used to prevent states of high tension: Eight patients (33%) talked about having tried to use DTS preventively before they got into states of high tension: *“[…] I have good experiences by now*, *um*, *with*, *with prophylaxis*, *like with using the skills in advance*. *[…] And it helps that I*, *I say like in 80% of the time*, *steer away just before the wall and don’t crash into the wall*.*”* (P9).

Subtheme D3.3. DTS are used automatically. Eight patients (33%) reported that they already used some DTS *“without thinking about it”* (P23) at the appropriate moment.

Subtheme D3.4. DTS make me feel safe. For 19 patients (79%), the DTS provided safety. In particular, they emphasized the advantage of being independent: *“Yes*, *to regulate states of tension independently*, *without needing help from the outside*, *so that I can help myself*. *Just to get back into a functional mode”* (P11), through which they get *“a piece of freedom”* (P3). The successful application of the DTS was perceived by some patients as a great achievement they can be proud of: *“Then*, *of course*, *in the end*, *the long-term joy of having pushed through and (…) been able to apply the skill and also helped*.*”* (P5).

Ten patients (42%) indicated that they already felt safer knowing that they could use DTS when needed: *“I know I have options available to help me cope in difficult situations and that gives me a certain amount of safety”* (P15). They reported to know about the effectiveness of DTS *“Yes*, *I did that back then*. *It worked well; so now I’ll do it again”* (P16) and therefore worry less when feeling tense: *“If it is automated you can*, *you can be in*, *like*, *states of high tension once in a while because if it is automated and the skills work”* (P23).

Subtheme D3.5. I can participate more in life. For 12 patients (50%), the long-term effect of DTS was being able to become part of society: *“That is really nice*. *Then you’re totally involved again*. *You’re attentive*, *you get*, *um*, *you can process the information that is said*, *well*. *And that is just a nice feeling*. *You*, *you are like*, *well it seems to me as if I am part of the cycle again*. *Um*, *that’s nice*.*”* (P7). The patients talked about being more involved: *“I got married in the meantime*. *I have a great husband*. *[…] even at work it’s going reasonably well*.*”* (P5). One patient stated that this changed their perspective on life: *“The fact that you have more joy in life in […] And on the nice days you can participate more in life*, *because you can*, *like*, *regulate yourself*.*”* (P23).

Subtheme D3.6: Better coping in difficult situations. Ten patients (42%) reported that they react less intensely in stressful situations and keep the consequences of one’s own actions in mind: *“Well*, *I think in order to avoid impulsive behavior*. *[…] the DTS just cause you to take a little distance and then*, *like*, *make it possible to think about how to deal with the situation in any other way*.*”* (P10). Patients reported that they could take more time to think before they react in stressful situations: *“Yes*. *Just let it sink in and sleep on it a few times and think about it in peace and quiet and not directly from 0 to 100*.*”* (P16) and as a result of this changed attitude, some patients noticed that the cause itself seemed less critical: *“And then it usually feels quite different with the trigger*, *that it might not be as bad as you first thought*.*”* (P5). Patients felt that this coping with difficult moments *“simply adds to the quality of life*.*”* (P13).

*Theme D4*: *The effect of Distress Tolerance Skills changes over time*. Five patients (21%) commented on changes in the effect of DTS during the course of therapy. They pointed out that the effectiveness of DTS decreases with frequent use and new DTS need to be sought: *“Well*, *you know how they say*: *‘skills wear out’*. *You*, *like*, *always have to be looking for somewhat different things”* (P15). For some patients the new skill had to be more intensive than their predecessor. One participant reported that, over time, weaker stimuli also became effective in states of high tension: *“Like*, *I don’t need such strong stimuli anymore*. *[…] I’m*, *like*, *practicing mindfulness*. *That’s also working well at higher levels of tension now*.*”* (P21).

*Theme D5*: *Distress Tolerance Skills are the first step in therapy*. Fourteen patients (58%) reported DTS to be the start of the therapy process, which enabled the next steps. Patients’ statements could be linked to three subthemes.

Subtheme D5.1. Without problem behavior I can focus on other topics. One patient frequently emphasized that when problem behavior occurs, it was treated as a priority in therapy: *“So*, *if I’ve been drinking or something*, *then the next therapy session will definitely be about a behavior analysis”* In order to break this cycle this patient used DTS instead of problem behavior (P24).

Subtheme D5.2. The therapy can’t reach me when I am in states of high tension. Ten patients (42%) explained that the contents of the therapy didn’t reach them when they were in states of high tension. *“If I sit there with a tension of over 70*, *then it doesn’t really get to me”* (P10). Patients said that they first have to be properly present in order to learn new skills: *“So that’s how it is*: *With emotion regulation skills […] you should be in the here and now and get everything properly*. *And with stress-tolerance skills*, *you first off use them to be back in the here and now again*.*”* (P21).

Subtheme D5.3. DTS do not change the problem itself. Seven patients (29%) criticized that DTS only regulated distress in the short term, but that they *“did not help the problem as such”* (P17). The causes of the disorder are not treated by the DTS: *“These disadvantages simply are like that*, *like*, *what you experienced in childhood*, *that that’s just there and that this wound is there and it’s not being treated*. *So*, *I really feel that this is a disadvantage*.*”*

## Discussion

The aim of this study was to explore the experiences of patients with BPD with the technique of DTS. Our goal was to examine factors patients perceived as hindering and facilitating when engaging in, learning and applying DTS as well as their perception of the short- and long-term effects of DTS. A thematic analysis of patients’ statements resulted in four key domains reflecting the process patients experienced while (Domain A) engaging in DTS, (Domain B) learning DTS, (Domain C) applying DTS to (Domain D) perceiving the effects of DTS. Patients reported hindering and facilitating factors during the initial engagement, the learning process and with the application of DTS. Patients also experienced various long- and short-term effects that they linked to the usage of DTS. In the following we discuss results for each research question separately linking them to the key domains.

### Factors patients experienced as hindering during the initial engagement, the learning process and the application of Distress Tolerance Skills

Patients’ statements revealed several internal and external factors that hindered the initial engagement in DTS, the learning process and the application of DTS.

#### Initial engagement

All patients reported difficulties regarding the initial engagement in DTS. Patients did not want to use DTS in the beginning as they experienced it as unfair that they had to do DTS, could not bring up the motivation, found DTS ridiculous or did not see how DTS could help their problems. Experiences that “problem behaviors” are much faster, easier to perform and more reliable in reducing distress made it hard for them to be open to DTS.

#### Learning process

Almost all of the interviewed patients reported that the learning process was a long and strenuous one. Patients reported feeling alone and overwhelmed in the search for DTS that were suitable for them out of a wide range of possibilities. Moreover, they found it hard to decide which skill to use in which specific situation and how to perform it correctly. Due to states of high tension in therapy sessions, some patients reported not being able to capture all of the information on DTS. Barnicot, Couldrey et al. [9] also found that participants reported feeling overwhelmed by getting a lot of information in a rapid pace during skills training and that patients felt reluctant towards skills use at times. Even if patients managed to engage in DTS, more than half of the patients in this study reported difficulties during the learning process.

#### Application of DTS in everyday life

More than half of the interviewed patients reported problems with not having access to their DTS during their day. They tended to forget their DTS at home; they couldn’t transport them or rated them as unsuitable for their everyday life. Interpersonal situations were reported to be especially distressing by most of the patients. However, almost all of the patients reported reluctance to use visible DTS in front of other people due to the fear of being judged or burdening others. Patients also reported that states of high tension caused them to forget about DTS, even if they had them on hand. This is in line with the findings of Barnicot et al. [9], who also identified high tension as a hindering factor for the use of skills. A related problem the patients encountered was that some DTS didn’t work in states of high tension, such as fine motor skills or cognitive skills and that they needed intense sensory stimuli. However, as sensory perception is reduced in states of high tension, some patients noticed a risk in using strong sensory stimuli (e.g. temperature, spiciness, ammonia) excessively or in a potentially harmful way.

### Factors patients experienced as facilitating during the initial engagement, the learning process and the application of Distress Tolerance Skills

#### Initial engagement

More than half of the patients reported having been motivated to engage in DTS by their desire for change regarding their problem behavior and their level of tension. These goals are in line with the findings of Katsakou et al. [15], who found that the therapy goals of patients with BPD included a reduction in problem behavior and an improvement of coping with emotions. Patients stated that the group therapy sessions helped them to accept DTS: Meeting others struggling with the same problems had a normalizing and validating effect on them. Learning about other patients’ perspectives on DTS and their positive experiences with the use of DTS helped them with the initial engagement in the technique. These validating and normalizing experiences during group sessions were also described as helpful in various other qualitative studies on DBT [8]. Moreover, patients in our study reported the importance of getting information about the aims, strengths and limitations of DTS in advance.

#### Learning process

Patients found it important for the learning process that they were able to estimate their level of tension. Some patients even used DTS to assess their level of tension as they would notice the reduced perception of sensory stimuli in states of high tension.

One third of the interviewed patients also appreciated the joint practical exercises and experiences with DTS during group sessions. Moreover, patients in our study underlined that the adaption of the pace to the individual patient was important. Patients also mentioned illustrative examples to be helpful in learning and remembering DTS. Barnicot et al. [9] also found that patients reported learning to be easier when there was a fun and light-hearted atmosphere and Cancian et al. [16] identified metaphors and exercises as being helpful in learning and remembering skills for women with obesity receiving a DBT adapted skills training.

A stable therapist-patient relationship was valued by patients with the therapists providing respect and understanding as well as clear guidance and expert knowledge. Patients’ experiences might reflect one of the most important features of DBT, the dialectic of acceptance and change within the therapeutic relationship [17]. Several other qualitative studies described similar patient reports: Little et al. [8] found reports about the relevance of the therapeutic relationship in several studies highlighting especially the importance of the therapists’ attitudes towards the clients and the therapist’s knowledge. In a meta-synthesis on qualitative studies of BPD patients’ experiences of treatment being cared for and respected by the therapist as well as the therapist focusing on change was valued as helpful [18]. Levitt et al. [19] found that both authentic care and structure in the therapist-client relationship was considered as important by patients receiving various kinds of psychotherapy.

#### Application of DTS

As most of the patients interviewed experienced high tension in interpersonal interactions and were reluctant to use DTS that could draw attention, they reported small and unobtrusive DTS to be helpful. The automatization of DTS use was regarded as necessary by the patients in order to have access to DTS in case of emergency and states of high tension. The process of the skills becoming automatic was also discussed in other studies: Little et al. [8] summarized in their meta-synthesis that patients reported skills to have become “ingrained” [9] and a “second nature” [20].

### Experienced effects of Distress Tolerance Skills

#### Short-term effects

All patients mentioned the reduction of tension as a short-term effect. The reduction of tension would help patients to be able to function again, bring them back from a dissociative state and help to prevent problem behavior. In some patients, the reduction of tension would lead to unwanted emotions immediately after the use of DTS. Patients regarded DTS as a first step in therapy: they reported the reduction of tension due to the use of DTS as crucial for being able to follow therapy sessions and to focus on important personal topics in therapy.

However, some patient argued that DTS only regulate distress in the short term, but that they did not “help the problem as such”. These experiences of patients match with the original idea of crisis survival skills by Marsha Linehan as ‘short-term solutions to painful situations. Their purpose is to make painful situations more tolerable, so that it is possible to refrain from impulsive actions that can make the situation worse’ [1]. ‘These skills are not a cure for all problems in life […].They are not designed to be emotion regulation strategies, although they may help to regulate emotions’ [1].

#### Long-term effects

Almost all patients reported feeling more stable and relaxed. They reported that DTS had helped them to gain insight into and acceptance of their problems. They also mentioned that they would get into states of high tension less often and would therefore need to use DTS less frequently. Some patients reported being able to use mindfulness skills instead of DTS in the long term. Many patients felt that the DTS-training had equipped them with tools to survive their everyday life which made them feel safe and self-confident. Half of the patients reported an increase regarding their quality of life and participation. This is in line with the results of the meta-synthesis of Little et al. [8], finding that patients receiving DBT highlighted the development of self-efficacy, learning skills to manage emotions as well as taking ownership and responsibility. Also, the long-term effects experienced by our patients partly overlap with the results emerging from a meta-synthesis of 14 qualitative studies on BPD patients’ experiences of treatment and recovery [18]. Findings suggest that clients experience changes in four main areas (with three of them in line with our results): self-acceptance and self-confidence, controlling difficult thoughts and emotions, practicing new ways of relating to others, and implementing practical changes and developing hope.

### Limitations and strengths

To the best of our knowledge, this is the first qualitative study exploring BPD patients’ experiences with DTS in detail. In this study more female than male participants were interviewed and no participants that had already dropped out of treatment at the time of the interview agreed to participate in the interview. One participant dropped out of treatment at a later stage of treatment. Also, due to the setting at a university clinic, the participants in this study were complex BPD patients with high BPD symptom severity and multiple comorbid disorders. Because of these reasons, generalizability could be limited.

Interestingly, patients in our study mainly described the use of crisis survival skills and omitted reality acceptance skills. There are various possible reasons for this. Firstly, it might be that crisis survival skills were more prominently discussed by therapists and that therapists did not pay enough attention to teach and have patients experience reality acceptance skills. Also, it might be that patients did take notice of information on reality acceptance skills but that crisis survival skills were remembered better. This might be the case especially for BPD patients with high BPD severity as were included in this study. Those patients might have had frequent crises which make crisis survival skills more important to them initially. Furthermore, crisis survival skills might have been perceived as applicable in a more active way than reality acceptance skills. They are associated with the experience of an instant reduction in tension which may make them more memorable. Also, the focus during the qualitative interviews could have unintentionally been directed towards a subgroup of DTS. Of course, it’s also possible that a combination of the above-named reasons has led to the omittance of reality acceptance skills.

Remarkably, patients in our study reported sensory skills like ammonia or spiciness more frequently than TIP-skills like “cold water” described in the original manual by Linehan [1] (TIP skills can be used to change body chemistry quickly, so as to counteract disabling emotional arousal. TIP is mnemonic for Temperature, Intense exercise, Paced breathing, and Paired muscle relaxation). The German adaptation of the Skills Training Manual could therefore differ from the original.

Also, it is important to keep in mind that the participants in this study learned DTS in the context of a DBT treatment lasting 18 months. Therefore, reported effects that were linked to DTS by the participants could also be related to other DBT techniques and the attribution of specific effects to specific techniques delivered in the context of a complex treatment program is difficult. In the analyses conducted in this article, we did not differentiate between participants’ statements on group therapists and individual therapists. This differentiation could have gained even more information regarding facilitating and hindering factors of therapists’ behavior in group and individual sessions.

The inclusion of the participants in this study was based on convenience sampling as the recruitment of participants was challenging because of limited reachability and willingness to participate. Therefore, it is possible that selection bias occurred, for example, because of the fact that participants that had dropped out of the treatment did not take part in the interview but also because of the fact that participants with higher symptom severity or participants that did not benefit from or were discontent with DBT were less likely to agree to participate in the interview.

A strength of this study was the consensual analysis of 21% of the interview material by two raters that had not been involved in the treatment, as well as the discussion of the category system within an expert group. The sample size of 24 patients seems to have fostered theoretical saturation.

### Clinical and research implications of the findings

Because of the subjective nature of qualitative findings and the above-mentioned limitations, the present study can only raise tentative suggestions for clinical implications and hypotheses for testing in further research.

#### Clinical implications

Based on the statements of the participants of this study, therapists can keep in mind several points to optimize future delivery of DTS training for patients with BPD:

1. Information about DTS in advance: In order to prevent misunderstandings and disappointment, therapists can point out strengths and limitations of DTS in advance. In our study, patients experienced it as helpful to know that DTS were only a tool for the moment and not the solution for the underlying problem. They also appreciated knowing about upcoming difficulties and the fact that DTS use would become easier with training and might eventually become less necessary as patients would proceed in therapy.
2. Stable therapeutic relationship: The patients in our study valued assertive therapists and a clear communication during DTS training as well as an environment based on validation and respect. Patients reported that this helped them to open up and feel free to ask questions.
3. Address difficulties with and reluctance to DTS use early: Most patients reported difficulties with learning and using DTS as well as reluctance to use DTS in the beginning of therapy. Therapists can therefore pay close attention to their patients’ problems, validating and normalizing difficulties and reluctance and help them overcome them. Barnicot, Couldrey et al. [9] suggested rating hindering factors for the use of skills as “treatment-interfering behaviors” and be treated with high priority and monitored using diary cards.
4. Use states of high tension as a chance to train DTS directly: Therapists should keep in mind that in states of high tension, where it is most important to use DTS, patients have difficulties to perform DTS. These difficulties range from not recognizing that their tension is high, over not having “DTS equipment” with them, to not remembering to use DTS or to feeling ashamed to use DTS. Thus, therapists should pay close attention to patients’ current state of tension, give them feedback using their observations and help them rate their level of tension regularly, as well as help them to reduce their level of tension during therapy sessions. They might encourage patients to have their “DTS equipment” with them or even better teach them skills they can use everywhere without equipment. A state of high tension during a therapy session is a chance to provide patients with the experience that DTS really work and help. As such, therapists should frequently directly train DTS with practical exercises in individual or group sessions, especially when patients are in high distress. By this, patients do not only train and get accustomed to using DTS, but also the use of DTS is normalized, shame is reduced and the practical experiences help to consolidate DTS.
5. Keep in mind that patients in distress might not remember all of the information: Also, therapists should keep in mind that patients in high distress are not receptive to therapy content. Therapists can therefore use the individual sessions to repeat the information learned during group therapy as patients’ level of tension might be lower during individual sessions. The joint application of a DTS at the beginning of group- and individual sessions could help bring down the level of tension into a range in which patients are receptive to therapy content. Barnicot et al. [9] also identified high levels of tension as a hindering factor for the use of skills and suggested to focus on the communication of mindfulness skills or on skills that include sensory stimuli like muscle relaxation.
6. Adjust to the pace of the patient: Therapists could keep in mind that DTS-training involves a lot of information, jargon and abbreviations that are difficult to follow and remember, especially in states of high tension. They can therefore pay close attention, repeat important content and help patients to feel free to address questions and uncertainties.
7. Usage of examples and practical training: The patients in our studies appreciated illustrative examples and practical training during individual and group sessions as this helped them to remember, try and integrate DTS. Cancian et al. [16] proposed that therapists could develop and use stories, metaphors and practical exercises for each taught skill in order to promote memorization and the learning of skills.
8. Help patients develop suitable DTS: Patients in our study highlighted the importance and difficulty of developing DTS suitable for their individual situation. In accordance with the tasks for DBT individual treatment from the original Linehan manual [1], therapists should use individual sessions to help patients tailor the DTS learned during group sessions to their everyday life. Therapists should keep in mind that most patients need DTS in interpersonal situations while experiencing DTS use in these situations as especially difficult because of their fear of being judged or burdening others. Thus, therapists could help patients to develop and practice DTS suitable for interpersonal situations and encourage them to use them on a regular basis. Also, patients in this study reported the need to automatize DTS in order to be able to apply them during everyday life and emergencies. Therapists could keep this in mind and encourage patients to practice DTS until they become automatized reactions.
9. Reduce overload: Patients in our study reported feeling overwhelmed by the wide range of DTS and the need to try out many DTS in order to identify suitable ones. Other studies found that patients are also overwhelmed by the presentation of the skills training material as it includes a lot of information and uses jargon and acronyms [9, 20]. Therapists should therefore adapt their pace and the amount of information they provide during one session to the individual patient, remember to check with the patient if they understand everything and ask if they need help in selecting and implementing suitable DTS.
10. Watch out for misuse of DTS: Some patients in our study reported being at risk of using DTS in an excessive way. Especially in states of high tension, strong stimuli can be necessary, such as spiciness or temperature or sports that can have harmful effects or turn into a problem behavior. In line with Linehan’s warnings that ‘crisis survival skills can amount to avoidance of building a life worth living’ [1] patients in our study also pointed out that DTS can be used to avoid emotions altogether, preventing adequate emotion regulation strategy and progress in therapy. Therapists therefore should monitor DTS use and address possible harmful or excessive use of DTS early.
11. Be clear about Crisis Survival Skills and Reality Acceptance Skills and ensure usage of both sets of DTS: Patients in our study mainly reported the use of crisis survival skills. As discussed earlier, this could be due to the fact that crisis survival skills feel more important or are more memorable because of their instant effect on tension. However, it could also be that Reality Accepting Skills are perceived as more difficult to apply by therapists and patients and therefore receive less focus in therapy. Therapists should teach both subgroups of DTS and help patients understand the dialectic of mindfully accepting arousal without judgment and acting to reduce arousal at the same time [21].

#### Suggestions for further research

Qualitative research in the field of DBT is still in its infancy and there are plenty of questions to be addressed. In future research, it is important to investigate the experiences of patients who dropped out of treatment as this might help to optimize DBT according to their needs. Moreover, besides assessing general experiences of BPD patients with DBT as a whole, future research would profit from a narrower focus and more specific questions on specific DBT modules, skills or therapeutic techniques. However, it might be a challenge to directly link patients’ experiences to a specific DBT module, skill or technique when offered in a complex DBT-treatment, like in our study where we cannot directly link the reported effects to DTS. Yet, offering DTS alone would not fit with the DBT rationale. Conducting qualitative interviews after each skill training module could shed more light on the perceived effects of each skill training component.

This analysis was one of the first of a qualitative research series we conducted on components of DBT. While we started with analyzing one module of skills training, namely DTS, in detail, it is important to analyze other components and modules of skills training in DBT as well, such as mindfulness, interpersonal effectiveness and emotion regulation. This can help identify specific mechanisms that facilitate or hinder the recovery process within the treatment, and help to gain further insight for practicing therapists regarding facilitating factors in the teaching of skills.

Also, in our study patients referred almost exclusively to crisis survival skills and did not share their experiences with reality acceptance skills. Future research should investigate if this is a matter of the qualitative assessments, therapists providing less information on reality acceptance skills, or of patients not remembering this information. Learning about the experiences of patients and therapists with reality acceptance skills could shed more light on possible difficulties and benefits regarding these DTS.

Interestingly, in our study, only one third of the patients reported to use DTS as an anti-dissociative technique and only one third mentioned the risk of DTS being used to avoid emotions. Future studies could focus on these aspects more thoroughly.

Further, as the reduction of tension seems necessary in order for patients with BPD to be perceptive of the therapy content, future research could investigate if the joint application of DTS at the beginning of every session from the start of DBT treatment could help to reduce the level of tension of patients during therapy sessions and if a reduced level of tension during therapy sessions is associated with therapy progress and symptom reduction.

## Conclusion

Patients with BPD experience various positive effects of DTS including an immediate reduction of distress, prevention of ‘problem behaviors’, anti-dissociative effects and becoming capable to function again. Patients perceived DTS as a tool to handle difficult situations and emergencies, which helped them to feel stable, safe and self-confident enough to enter situations they previously avoided and therefore help generate improvement in various areas of patients’ lives. However, patients experienced the training and usage of DTS as difficult and overwhelming and had problems with the acceptance of DTS in the beginning of treatment. Information, validation and support in developing and automatizing individually suitable DTS, as well as dealing with states of high tension early in sessions can help patients to overcome their problems with and reluctance towards DTS. Hindering and facilitating factors during the initial engagement, the learning process and the application of DTS that were identified in this study could help therapists to optimize DTS-training. If mastered, DTS present an important first step in the therapeutic process as they provide a powerful way to reduce distress, help the patient become receptive to therapy content and can support the above-mentioned important changes in the patients’ lives.

## Supporting information

S1 File Qualitatives interview zu Elementen der DBT–Teil 1 (German original). (DOCX)
