# Supplementary material for: How do patients with borderline personality disorder experience Distress Tolerance Skills in the context of dialectical behavioral therapy?—A qualitative study
Source: PLoS One. 2021 Jun 15;16(6):e0252403. doi: 10.1371/journal.pone.0252403 (PMC8205175; doi:10.1371/journal.pone.0252403)
Supplement: S1 File — (DOCX) [file pone.0252403.s001.docx]

| Wir konzentrieren uns jetzt zunächst auf die Stresstoleranz-Skills.   1. Nennen Sie die für Sie wichtigsten Stresstoleranz-Skills (max. 5) 2. Welche Erfahrungen haben Sie bisher mit der Anwendung von Stresstoleranz-Skills gemacht? 3. Wie häufig setzen Sie Stresstoleranz-Skills in etwa ein? *(z.b. mehrmals am Tag, 1x/am Tag, 1x/pro Woche...)* 4. In welchen Situationen tun Sie das? 5. Welche Effekte bemerken Sie, wenn Sie Stresstoleranz-Skills einsetzen? (*günstige und ungünstige Effekte, kurzfristig und langfristig?)* 6. Wie zufrieden sind Sie mit der Wirkung von Stresstoleranz-Skills? 7. Wie erklären Sie sich die Wirkung von Stresstoleranz-Skills bei Ihnen? 8. Was sind hinderliche Faktoren für Sie, Stresstoleranz-Skills einzusetzen? 9. Was unterstützt Sie dabei, Stresstoleranz-Skills einzusetzen? 10. Gibt es etwas das Sie den Therapeuten sagen möchten, die Stresstoleranz-Skills vermitteln?   Gab es etwas das Sie als hilfreich oder nicht hilfreich empfunden haben?  Gab es etwas, dass Sie sich noch gewünscht hätten? |
| --- |
